# On the time course of short-term forgetting: a human experimental model for the sense of balance

**DOI:** 10.1007/s11571-015-9362-0

**Published:** 2015-11-07

**Authors:** Arne Tribukait, Ola Eiken

**Affiliations:** Department of Environmental Physiology, Swedish Aerospace Physiology Centre, School of Technology and Health, Royal Institute of Technology, KTH, Berzelius väg 13, 171 65 Solna, Sweden

**Keywords:** Short-term memory, Spatial memory, Spatial orientation, Vestibular memory, Vestibular psychophysics

## Abstract

The primary aim of this study was to establish whether the decline of the memory of an angular displacement, detected by the semicircular canals, is best characterized by an exponential function or by a power function. In 27 subjects a conflict was created between the semicircular canals and the graviceptive systems. Subjects were seated, facing forwards, in the gondola of a large centrifuge. The centrifuge was accelerated from stationary to 2.5G_z_. While the swing out of the gondola (66°) during acceleration constitutes a frontal plane angular-displacement stimulus to the semicircular canals, the graviceptive systems persistently signal that the subject is upright. During 6 min at 2.5G_z_ the perceived head and body position was recorded; in darkness the subject repeatedly adjusted the orientation of a luminous line so that it appeared to be horizontal. Acceleration of the centrifuge induced a sensation of tilt which declined with time in a characteristic way. A three-parameter exponential function (Y = Ae^−bt^ + C) and a power function (Y = At^−b^ + C) were fitted to the data points. The inter-individual variability was considerable. In the vast majority of cases, however, the exponential function provided a better fit (in terms of RMS error) than the power function. The mean exponential function was: y = 27.8e^−0.018t^ + 0.5°, where t is time in seconds. Findings are discussed with connection to possible underlying neural mechanisms; in particular, the head-direction system and short-term potentiation and persistent action potential firing in the hippocampus are considered.

## Introduction

In its more primitive appearances the nervous system—or ganglion—is obviously devoted to stereotypic responses to sensory stimuli (Swanson 2000). The evolution of the vertebrate brain seems to have started with decussating inter-neurons, mediating the curling response in amphioxus (Sarnat and Netsky 2002). It also appears that the predictability of responses decreases with increasing complexity, e.g. the level of neural circuitry (Katz 2007), of the brain. Presumably, this stimulus-independency is related to the development of memory systems and conscious thinking. Even if an entity such as consciousness would never become available to objective empirical investigation (cf. Fabbro et al. 2015), attempts to discern its main components, and physical basis, have produced a plethora of imaginative theories (for notable contemporary examples, see Taylor 2007 or Werner 2009). The concept of memory appears to be as persistent as it is central for the understanding of complex species; dating back to the ancient Greeks (for a review, see Danziger 2008) it prevails in most branches of psychology and neurobiology.

Accordingly, the word ‘memory’ is applicable to a vast number of phenomena, related to the brain or psyche (Cassel et al. 2012; Squire 2004). Nature’s purpose with a specific memory system would be reflected not only in the characteristics of the stored information but also in its temporal properties and in the sensitivity to various interfering factors. One fundamental question is how cognitive processes or memory phenomena on the psychological level are related to biophysical mechanisms (Mizraji et al. 2009). To explain experiential phenomena by processes on neural level is, however, an endeavour with inescapable difficulties; mental states cannot be reported by animals whereas neuronal activity cannot be directly recorded in healthy human subjects.

A few related questions concern the nature of *forgetting*. For instance, which are the causes of forgetting (Nairne 2002; Ricker et al. 2014) and what is the *time course* of forgetting? The mathematical characteristics of psychological forgetting, as well as of decay phenomena in neural tissues, might give hints regarding how processes on different levels are interrelated (cf. Tarnow 2008, 2009). To establish the time course of forgetting requires the identification of a quantifiable phenomenon related to a well-defined initial stimulus. A typical example is the recollection of a number of items, or words, after a period of time. Naturally, the number of items recalled will be smaller after a larger retention interval. The outcome of such experiments is, however, dependent on factors that are difficult to control. The retention of visual and auditory stimuli engages the so-called working memory (Baddeley 1986) and it is, thus, dependent on conscious strategies or activities of the test subject (cf. Logie et al. 1990; Makovski and Jiang 2008; Pellegrino et al. 1976; Silverberg and Buchanan 2005). Further, since a memory trace is also likely to be influenced by the subject’s reporting, several measurements after one and the same stimulus will not reflect a neutral forgetting process. Therefore, data for different retention intervals have to be obtained separately using different—but equivalent—stimuli. These, and several other, circumstances make the collection of data and the mathematical characterization of memory decay rather intricate (for an overview of the problems, see Rubin and Wenzel 1996).

The mathematical form of forgetting—from short-term as well as from long-term memory—remains a matter of lively debate, a crucial choice being that between an exponential function, y = Ae^−bt^, and a power function, y = At^−b^ (where A and b are constants and t represents time). The exponential function is attractive because many basic processes in nature display an exponential time course (cf. Murdock and Cook 1960). Nevertheless, within psychology the power function has since long possessed a certain status—not only in laws regarding the perception of stimulus intensity (Stevens 1970), but also when it comes to characterising training effects (several examples are given by Newell and Rosenbloom 1981), and forgetting (e.g. Wixted and Ebbesen 1991).

The controversy regarding the form of forgetting is probably to some extent due to the fact that there are several memory systems, each of which can be studied in different ways. In addition, the procedure for data treatment can, in itself, become a problem. For instance, if there is a substantial inter-individual variability in the time constant for a truly exponential memory decay, then curve fitting to group data may generate an *artifactual* power function (Anderson 2001; Anderson and Tweney 1997; Murre and Chessa 2011). More generally, pooling data from different individuals—or from different experiments—may result in forgetting curves that are not representing real mental or neural processes.

An analogous line of reasoning concerns the relationship between psychological memory phenomena and underlying neural processes. Thus, the temporal characteristics of forgetting may reflect the sum of two or more neurobiological mechanisms with different decay functions. As noted by Sikström (1999), if performance in a memory task were dependent on multiple memory traces, decaying exponentially with different time constants, forgetting could proceed according to a power function even in the individual. Conversely, a single neuronal memory mechanism would more likely be reflected in the recording of a simple quantifiable impression not accessible to conscious manipulation. As regards visual stimuli, one elementary attribute, processed in the primary visual cortex, is the orientation of a line (Hubel and Wiesel 1977; Spillmann 2014). The ability to perceive the orientation of a visual object is, however, also dependent on information regarding the position and motion of the head with respect to the surface of the Earth.

*Thesense of balance* has a comparatively small cortical representation (Brandt and Dieterich 1999; Lopez et al. 2012). This “sixth sense” is generally regarded as a silent companion, the activity of which we become aware of mainly during abnormal stimulation or vestibular disorders (vertigo). The organ of balance comprises two receptor systems, the semicircular canals, responding to angular head movements, and the otolith organs, which sense linear accelerations as well as the orientation of the head in the Earth’s gravity field. On a central nervous level, the angular-*velocity* signal from the semicircular canals can be integrated over time, yielding an estimate of angular *displacement* (Clark and Taube 2012; Israël et al. 1996; Mergner et al. 1996). Changes in head orientation relative to gravity often generate redundant information; the brain receives transient signals from the semicircular canals, responding to the movement *per se*, and from the otolith organs, sensing the head’s position with respect to gravity prior to and after the movement. Our conscious perception of the visual world as upright and stable also during head tilt or head movements is the manifestation of compensation processes based on information from the vestibular organ (Bischof 1974; Petrov and Zenkin 1973).

A basic measure of spatial orientation is the subjective visual horizontal (SVH) or vertical (SVV) (Aubert 1861; Mittelstaedt 1991; Müller 1916; Van Beuzekom et al. 2001). This can be recorded using an adjustable luminous line in darkness; by adjusting the line the test subject indicates what he or she perceives as horizontal (or vertical). In vestibularly healthy individuals, sitting upright, the deviation from veridicality is rarely more than 2.5° (Dai et al. 1989; Tribukait 2006). Measurement of the SVH or SVV during static head and body tilt constitutes a test of otolith function (Dai et al. 1989; Mittelstaedt 1991; Tribukait 2006). Nevertheless, if a change in head orientation with respect to gravity is performed rapidly, then also the semicircular canals can have a significant influence on the SVH or SVV (Stockwell and Guedry 1970; Tribukait and Eiken 2006a, b; von Holst and Grisebach 1951; Udo de Haes and Schöne 1970). Although recording of the SVH or SVV is mainly regarded as a test of the sense of balance (cf. Mittelstaedt 1991), it makes use of the precision of conscious vision. Thus, processes inaccessible to conscious reasoning can be studied with great accuracy.

**Fig. 1:**
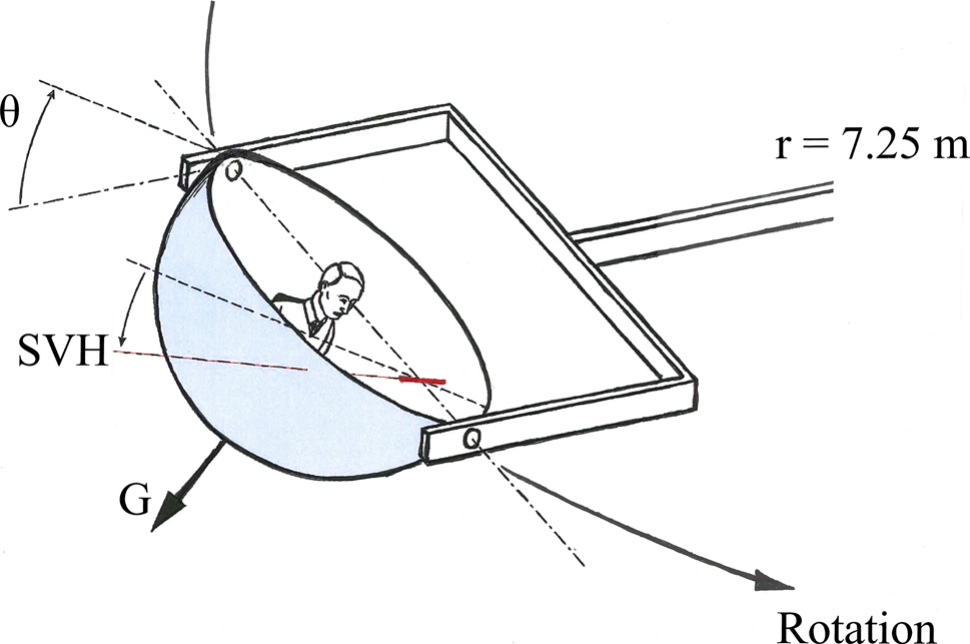
Schematic illustration of the intra-vestibular conflict occurring in a swing-out gondola centrifuge. During acceleration of the centrifuge, the gondola is rolled so that the test subject remains upright with respect to the G vector (resultant of the Earth’s gravity force and the centrifugal force). Therefore, the graviceptive systems do not receive a roll-tilt stimulus. However, the inclination (θ) of the gondola and subject with respect to the Earth-horizontal plane is a roll angular displacement stimulus to the semicircular canals. Recording of the SVH via adjustment of a *luminous line* in *darkness* provides a measure of how the subject perceives this angular displacement

Using a large swing-out gondola centrifuge (Fig. 1) it is possible to create an *intra*-*vestibular conflict* regarding head position. For instance, if the test subject is seated upright and heading forwards, acceleration of the centrifuge from stationary to 2G will create a 60° angular displacement in the roll (frontal) plane, similar to that occurring when tilting one’s head towards the shoulder (Glasauer 1993; Guedry et al. 1992). The increasing gravitoinertial force vector (vectorial sum of the centrifugal force and the force of gravity) is, however, persistently aligned with the head and body z (vertical) axis; hence, the message from the otolith organs is that the subject remains upright. In other words, a conflict arises between the canals, sensing a change in roll angular position, and the otoliths, persistently signalling for upright position (Tribukait and Eiken 2006b; Young 1984).

In a few recent studies the perceptual consequences of this canal-otolith conflict have been investigated via measurement of the SVH (Tribukait and Eiken 2006b). It has turned out that a great majority of the subjects make considerable under-estimations of the tilt angle. In addition, the perceived roll tilt angle declines with time, approaching zero within a few minutes. Nevertheless, this decline in perceived roll tilt follows a considerably slower time course than per-rotatory canal phenomena, i.e. nystagmus and perceived angular velocity (Tribukait and Eiken 2006b). Thus, it must represent a memory for canal information on angular changes in head orientation.

It occurred to us that this test paradigm might be useful for establishing the mathematical time course for short-term memory processes. Namely, there is a brief and well-defined stimulus (roll tilting of the gondola), the fading memory of which may be recorded as a continuous variable with the same unit (°). The simplicity of the recording (intermittent adjustment with a luminous line in darkness) implies that there is no feedback to the subject and, hence, no memory consolidation. In other words, since an adjustment of the line seems unlikely to influence the following adjustments, numerous recordings may be performed at different points in time after one single stimulus. There is also a natural end point for the forgetting process, namely the SVH value determined by the graviceptive systems, which in normal subjects is usually close to zero. Finally, the magnitude of the gravitoinertial force vector (G level) may be considered as an interfering factor that is also quantitatively definable.

The present study was part of a research programme regarding the effects of graviceptive input on semicircular canal phenomena. To permit adequate comparisons of data obtained in different stimulus conditions, and also to facilitate the identification of underlying neurophysiologic mechanisms, we wanted to establish the mathematical time course for decay of the canal-induced sensation of lateral tilt during gondola centrifugation. Thus, the study aim was twofold: Firstly, we wanted to compare the power function and the exponential function with respect to goodness of fit and discuss our findings in connection with the literature on the form of forgetting. Secondly, we wanted to consider some mechanisms and decay phenomena at the neurophysiologic level, which could possibly be of significance for the phenomenon recorded in the centrifuge. These comprise the so-called head-direction system as a basis for the sense of direction as well as spatial cells and short-term neural processes in the hippocampus.

## Methods

### Subjects

Data from 27 healthy subjects (9 females and 18 males), aged 21–51 years, were included in the study. The subjects did not have any experience of manoeuvring an aircraft and they were not motorcycle drivers or athletes on a competitive level. The subjects participated with their informed consent and were free to withdraw at any time during the experiment. The test procedures were in accordance with the declaration of Helsinki and were approved by the human ethics committee in Stockholm.

### Centrifugation

The experiments were performed in the swing-out gondola centrifuge at KTH in Solna, Sweden. The radius of this centrifuge is 7.25 m. Rotation is anti-clockwise (as seen from above). The gondola is tangentially pivoted and deflects outwards in the direction of the resultant force vector (vectorial sum of the Earth gravity force and the centrifugal force) (see Fig. 1). Heading forwards, the subject was fixed in the gondola by means of safety belts and a head holder. The head was positioned so that a line from the external auditory meatus to the inferior margin of the orbit was tilted upwards (nose up) approximately 10° with respect to the gravitational horizontal. The inter-ocular line was gravitationally horizontal. The subject was observed in infrared light using a video camera and he or she was always able to communicate with the experimenter via a two-way intercom system. The subject’s heart rate and rhythm were monitored continuously by means of electrocardiography.

Data were obtained in two experimental series. In series A, 12 subjects (three females and nine males, aged 22–41 years, denoted subjects 1–12) were exposed to three different G levels (1.1G, 1.7G, and 2.5G). The subjects participated in two sessions with an interval ranging between 1 h and 6 days. In the first session the G levels were presented in ascending order, in the second the order was reversed. In series B, 15 subjects (six females and nine males, aged 21–51 years, denoted 13–27) were exposed to 1.1G and 2.5G in air as well as immersed in water to the neck. Five of the subjects (No. 13–17) in series B underwent the test twice with an interval of 1–7 days. In the present analysis we will use data obtained at 2.5G without water immersion. Data for subjects 1–12 have been included in an earlier publication (Tribukait and Eiken 2006b). An analysis of the effects of water immersion (subject 13–27) will be presented elsewhere.

The centrifuge was accelerated from stationary to 2.5G. At 2.5G the angular velocity of the centrifuge about its main axle is 101°/s and the frontal plane (roll) inclination of the gondola is 66°. The angular acceleration of the centrifuge was 15°/s^2^ in series A and 7°/s^2^ in series B; i.e. the 2.5-G level was attained within 7 and 14 s, respectively. Thus, in both series the mean frontal-plane angular velocity was well above the stimulus threshold for the semicircular canals. The recording time at 2.5G was 6 min. Pauses between runs were 20 min (minimum); during the pauses the gondola was opened and the light was turned on.

### Measurements of the SVH

In front of the subject there was a line of red light-emitting diodes subtending a visual angle of 6.5°. The line could be rotated about the subject’s naso-occipital (visual) axis. The subject used two push-buttons on a remote control to adjust the line, every time it was switched on, so that it appeared to be horizontal (i.e. coincided with the subject’s spontaneous imagination of the horizon of the external world). When pleased with a setting, the subject pressed a third button, which extinguished the line. The deviation of the line from the gravitoinertial horizontal was then recorded with an accuracy of 0.1°. Before the line was switched on again it was rotated 5°–20° (randomly), alternately clockwise and counter-clockwise with respect to the subject’s latest setting. Except for the line the gondola was completely dark.

The subject was instructed to imagine the horizon of the sea and to adjust the line so that it was parallel with this horizon. In case of any sensation of being tilted sideways, he or she should indicate the horizon in relation to which he or she felt tilted, not the transversal plane of the own head. The subject was encouraged to trust his or her own feelings rather than thinking and calculating.

Prior to centrifugation an initial series of 8 settings of the luminous line was performed. During centrifugation, data collection commenced (i.e. the line was switched on) as soon as the 2.5-G level was attained. The test subjects made 3–5 settings of the line per minute.

### Definitions and treatment of data

Tilt of the SVH to the right (right end of the line set down, from the subject’s point of view) is denoted positive; tilt to the left is denoted negative. For each subject, the 1-g value for the SVH was calculated as the mean of the 8 settings made in the beginning of the experimental session. When establishing the exponential time course for the decay of the SVH tilt, we used the function SVH = Ae^−bt^ + C, where C represents an asymptote. This third parameter is motivated by the fact that normal individuals, while gravitationally upright, often have a deviation (albeit usually <2.5°) of the SVH from the true gravitational horizontal (Dai et al. 1989; Tribukait 2006); at an increased gravitoinertial force vector, acting in parallel with the subject’s head-to-seat (z) axis, this component of the SVH can be even greater (Tribukait and Eiken 2006b). For the sake of comparability with respect to goodness of fit, we have used this third parameter also when fitting the power function. Least squares curve fitting was performed by means of Microsoft Excel Problem Solver, using the so-called multistart for global optimization. Assuming that the values of SVH have the same uncertainty irrespective of the point in time, each datum is given equal weight for curve fitting, which is the default procedure when uncertainties in y are unknown (Harris 1998). Curve fittings were performed for every single run. For individuals who underwent two runs, mean values for A, b and C, as well as for the root mean square (RMS) error, has been calculated prior to performing statistics on group level.

In order to see how pooling data from different subjects can influence the form of the forgetting curve, curve fitting to group data was performed in three different ways: (1) The 6-min recording period was divided into 15 equal time intervals. For the individual, the mean time and SVH value was calculated for each interval. Then, for the group, the mean time and SVH value was calculated for each interval. Curve fitting was performed to the resulting 15 data points. (2) The same procedure was employed with the recording interval divided into 5 equal intervals. (3) Exponential and power function fits were made to all individual data points (n = 921).

## Results

When the subjects were sitting upright in the 1-g environment, the SVH was close to the true gravitational horizontal. The group mean was −0.64° ± 1.14° (SD). The greatest deviation (−3.1°) was shown by subject 4.

**Fig. 2:**
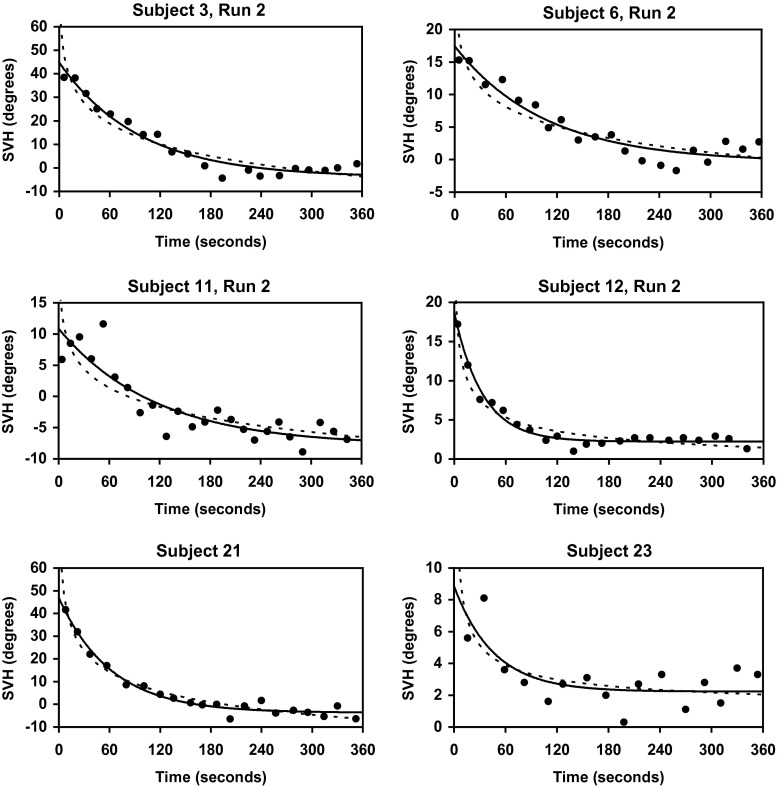
The SVH as a measure of roll tilt in six subjects. Each data point represents one setting of the *luminous line*. Time 0 is the beginning of the 2.5-G plateau. As a rule, the SVH tilt is considerably smaller than the real tilt of the gondola, and it decays with time, approaching an asymptote close to 0 by the end of the recording period. *Continuous lines* represent best-fitting exponential functions; *dotted lines* represent best-fitting power functions. Please, note that the vertical scales are different

Acceleration of the centrifuge induced a sensation of head and body tilt to the left. Consequently, the subjects adjusted the luminous line so that it was tilted to the right with respect to the horizontal plane of the gondola (i.e. the line was rotated clockwise from the subject’s point of view). This SVH tilt gradually decayed during constant angular velocity of the centrifuge. Data for six individuals are shown in Fig. 2.

Table 1 presents, for each individual, the best-fitting three-parameter exponential function. Corresponding findings for the power function are shown in Table 2. In addition to the constants A, b, and C, the extreme values assumed by the functions within the *observation interval* are also shown. Since none of the subjects made any settings of the line when t was <4 s, we have defined the observation interval as the time period between 4 and 360 s. This also makes it possible to compare the extreme values of the two functions, which would not be feasible for very small values of t, since the power function approaches infinity as t goes to zero from the right.

**Table 1:** Individual results of fitting (unweighted least squares sum) the three-parameter exponential function (SVH = Ae^−bt^ + C) to the settings of the line

Subject	A (°)		b (s^−1^)		C (°)		F(4) (°)		F(360) (°)	
1	45.7	30.3	0.0223	0.0101	7.7	5.6	49.6	34.7	7.9	6.4
2	17.6	31.7	0.0104	0.0439	6.6	3.0	23.5	29.6	7.0	3.0
3	41.8	48.8	0.0101	0.0105	−3.9	−4.0	36.2	42.8	−2.8	−2.9
4	36.3	18.3	0.0052	0.022	−13.2	−2.7	22.4	14.1	−7.6	−2.7
5	22.7	11.4	0.012	0.0334	1.2	3.3	22.9	13.3	1.6	3.3
6	15.4	17.8	0.0084	0.0094	4.8	−0.4	19.7	16.8	5.5	0.2
7	14.5	24.0	0.0057	0.0163	2.3	3.6	16.6	26.1	4.2	3.6
8	38.4	33.2	0.012	0.019	8.5	6.3	45.1	37.1	9.0	6.4
9	9.8	10.7	0.0058	0.0082	2.8	3.0	12.4	13.3	4.0	3.5
10	46.0	48.2	0.0146	0.0172	4.8	2.3	48.2	47.3	5.0	2.4
11	31.7	18.7	0.0034	0.0087	−16.5	−7.8	14.7	10.2	−7.2	−7.0
12	16.4	16.4	0.0154	0.0302	2.3	2.2	17.7	16.8	2.4	2.2
13	26.5	10.8	0.0665	0.0856	5.1	1.3	25.5	9.0	5.1	1.3
14	27.2	22.4	0.0108	0.0086	−1.0	−4.2	25.0	17.5	−0.5	−3.1
15	50.7	60.0	0.0289	0.0278	−3.1	−3.6	42.0	50.1	−3.1	−3.6
16	19.3	10.0	0.0424	0.051	2.7	2.0	19.0	10.1	2.7	2.0
17	60.5	67.8	0.0041	0.0022	−11.3	−25.8	48.1	41.2	2.5	4.9
18	22.4		0.0074		−0.5		21.3		1.1	
19	36.1		0.0142		7.1		41.2		7.3	
20	18.4		0.0124		4.4		21.9		4.6	
21	50.5		0.0161		−3.7		43.7		−3.5	
22	19.8		0.0146		7.0		25.7		7.1	
23	6.6		0.0216		2.2		8.3		2.2	
24	19.2		0.0118		7.6		25.9		7.8	
25	17.1		0.0057		−0.2		16.5		2.0	
26	47.2		0.0208		−2.8		40.6		−2.7	
27	11.7		0.0217		−0.0		10.7		0.0	
Mean	27.76		0.01805		0.49		26.49		1.99	
Median	22.39		0.0146		2.28		21.33		2.45	
SD	15.18		0.01468		6.10		12.70		4.10	

The extreme values of the function during the observation interval, i.e. at t = 4 s and t = 360 s, are also shown. For individuals who have undergone two runs (i.e. Subject 1–17), the means for run 1 and run 2 have been calculated before performing group statistics. Values of the constants are rounded in order to keep the table within reasonable limits; during the curve-fitting procedure the number of significant digits was >10 for each parameter

**Table 2:** Individual results of fitting (unweighted least squares sum) the three-parameter power function (SVH = At^−b^ + C) to the settings of the line

Subject	A (°)		b		C (°)		F(4) (°)		F(360) (°)	
1	112.2	3350.1	0.2256	0.0026	−24.7	−3294.5	57.4	43.5	5.0	4.7
2	4445.1	61.4	0.0010	0.4056	−4412.2	−4.4	26.7	30.6	6.8	1.3
3	4330.9	9509.6	0.0027	0.0013	−4265.4	−9439.3	49.3	53.2	−2.8	−2.2
4	6115.0	51.4	0.0012	0.1498	−6076.2	−25.4	28.6	16.3	−4.3	−4.1
5	4666.9	51.5	0.0013	0.7716	−4631.4	2.6	27.0	20.3	−0.1	3.2
6	84.0	3584.1	0.0891	0.0012	−44.5	−3558.0	29.7	20.1	5.2	0.8
7	9590.4	308.3	0.0004	0.0208	−9565.3	−270.8	19.8	28.8	2.5	2.0
8	9814.5	451.5	0.0010	0.0170	−9748.7	−404.4	52.2	36.6	8.2	4.1
9	4541.3	9547.8	0.0006	0.0003	−4521.3	−9527.1	16.2	16.7	4.0	3.8
10	9834.9	1827.9	0.0012	0.0062	−9763.8	−1763.3	54.8	48.9	1.9	−0.9
11	7757.7	9598.4	0.0008	0.0005	−7727.5	−9578.9	21.6	12.8	−6.2	−8.8
12	104.4	33.5	0.0472	0.3271	−77.7	−3.4	20.1	17.8	1.4	1.5
13	53.4	26.7	0.7321	1.0439	4.0	1.3	23.3	7.5	4.7	1.3
14	9671.1	9667.6	0.0006	0.0006	−9635.7	−9637.8	27.4	21.8	1.3	−4.3
15	105.2	122.9	0.2165	0.2169	−36.3	−41.4	41.7	49.6	−6.9	−7.1
16	32.6	18.9	0.4014	0.5932	−1.1	1.2	17.6	9.5	2.0	1.8
17	14,325	14,077	0.0008	0.0006	−14,247	−14,017	62.6	49.6	11.2	11.6
18	4792.0		0.0010		−4760.1		25.2		3.7	
19	14,577.3		0.0006		−14,521		44.6		5.3	
20	8776.1		0.0005		−8746.7		23.2		3.5	
21	169.1		0.1296		−85.3		56.0		−6.5	
22	6135.7		0.0008		−6102.3		26.7		4.6	
23	22.4		0.4738		0.7		12.3		2.0	
24	9211.9		0.0005		−9175.6		29.9		9.2	
25	9224.8		0.0005		−9197.3		21.1		0.4	
26	1906.9		0.0053		−1854.4		38.5		−6.1	
27	3900.3		0.0006		−3887.1		10.0		−0.5	
Mean	4913.1		0.1205		−4870.2		30.3		1.4	
Median	4792.0		0.0053		−4760.1		24.9		2.0	
SD	4236.3		0.2129		4236.5		14.5		4.7	

The extreme values of the function during the recording interval, i.e. at t = 4 s and t = 360 s, are also shown. For individuals who have undergone two runs (i.e. Subject 1–17), the means for run 1 and run 2 have been calculated before performing group statistics. Although the functional values for t = 4 s and t = 360 s were within reasonable limits, the power function tended to assume an asymptote (C) far beyond the physiological range. Values of the constants are rounded in order to keep the table within reasonable limits; during the curve-fitting procedure the number of significant digits was >10 for each parameter

In all 27 cases, exponential curve fitting gave values for initial and final SVH tilts of magnitudes similar to those really observed, corresponding to an initial tilt sensation smaller than the actual tilt (66°) and an asymptote close to 0°. Group means (±1 SD) for A and C were 27.8° ± 15.2° and 0.5° ± 6.1°, respectively. The time constant for exponential decay of the SVH tilt can be calculated as T = 1/b. The group mean for T was 85 ± 62 s.

Also the power function seemed to give a reasonable approximation within the observation interval (the period during which data points were obtained). However, the asymptote (C) was generally far beyond the physiological range, i.e. it would suggest that centrifugation in the long run causes a sensation of being rotated in roll (clockwise) by several hundred degrees (see Table 2).

As regards the RMS error, this was smaller for the exponential function than for the power function in the vast majority of cases (see Table 3). Group means for the RMS error were 2.416° ± 0.984° and 3.160° ± 1.537°, respectively (i.e. the RMS was 31 % higher for the power function). The difference between these two means is statistically significant (paired *t* test, *p* < 0.0001).

**Table 3:** The RMS error for each individual curve fit

Subject	Data points		RMS (°) exponential		RMS (°) power	
	Run 1	Run 2	Run 1	Run 2	Run 1	Run 2
1	19	18	3.085	2.304	4.089	2.058
2	20	17	3.596	1.846	4.019	1.496
3	17	20	3.414	2.765	4.233	4.376
4	16	18	4.265	1.487	6.037	2.018
5	16	19	2.802	1.374	3.349	1.593
6	17	20	1.375	1.545	1.267	2.095
7	27	26	2.121	1.780	2.560	1.576
8	21	24	3.504	2.935	4.061	3.748
9	14	18	0.943	0.595	1.102	0.835
10	21	24	3.849	3.278	5.207	5.196
11	21	24	1.812	2.427	3.177	3.134
12	21	22	0.796	0.626	0.963	0.934
13	27	26	1.479	1.515	1.578	1.646
14	24	24	3.069	1.880	4.286	2.419
15	20	24	2.918	3.774	5.023	5.703
16	23	23	1.915	1.830	2.379	1.911
17	24	23	3.477	3.442	6.869	6.726
18	16		2.136		3.135	
19	26		3.399		4.430	
20	22		1.671		2.135	
21	20		1.977		2.459	
22	19		3.004		3.531	
23	16		1.193		1.304	
24	15		4.474		4.648	
25	19		1.253		1.725	
26	20		4.151		5.898	
27	26		2.059		2.213	
Mean			2.416		3.160	
Median			2.136		3.074	

The number of settings of the line (data points) is also shown for each run. In only four cases (centrifuge runs) out of 44, the RMS was smaller for the power function than for the exponential function. It can also be noted that the RMS was considerably smaller than the change in SVH during the recording interval (see Table 1; Fig. 2). For individuals who have undergone two runs, the mean RMS for run 1 and run 2 has been calculated before performing group statistics

**Fig. 3:**
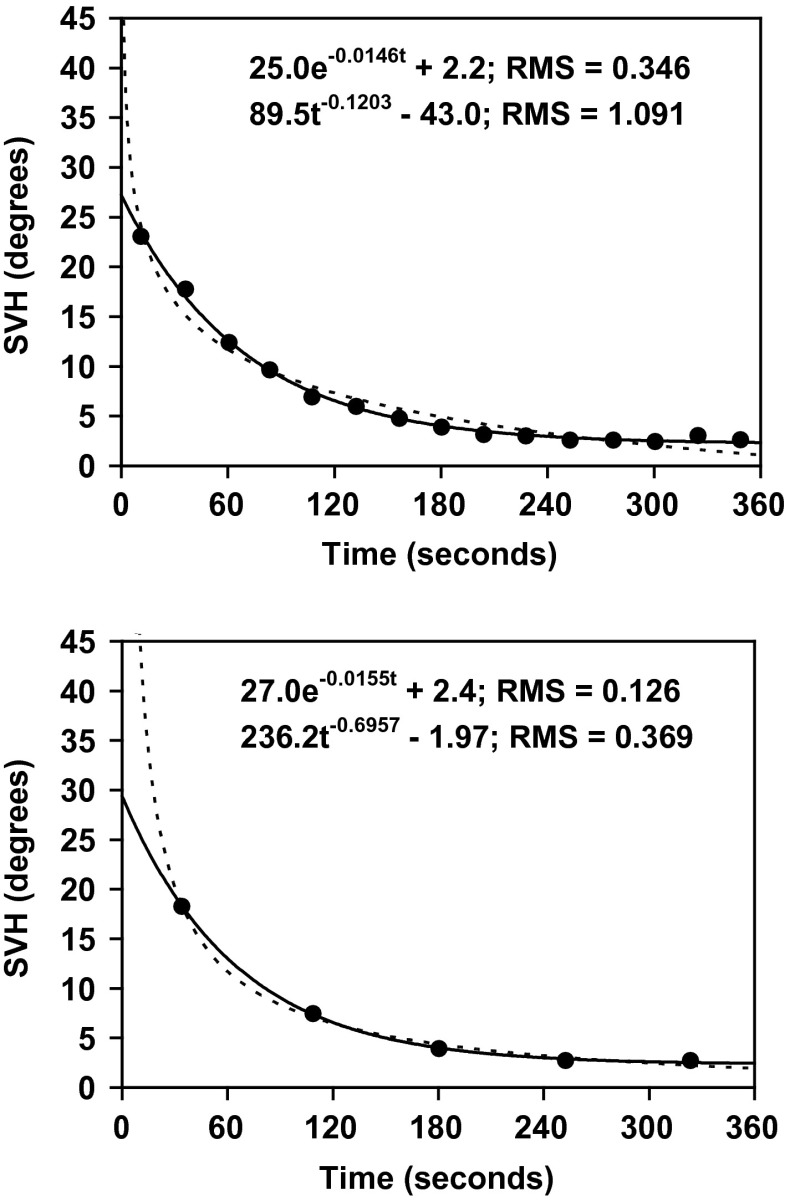
Adaptation of the exponential function (*continuous lines*) and the power function (*dotted lines*) to group data. The recording period has been subdivided into 5 or 15 equal time intervals. For the individual, a mean value (for time as well as for the SVH) has been calculated for each interval. Then, group means have been obtained in a corresponding way. To the unaided eye both curves might appear to be adequate fits to the data points. Nevertheless, in both cases the RMS error was considerably smaller for the exponential function

**Fig. 4:**
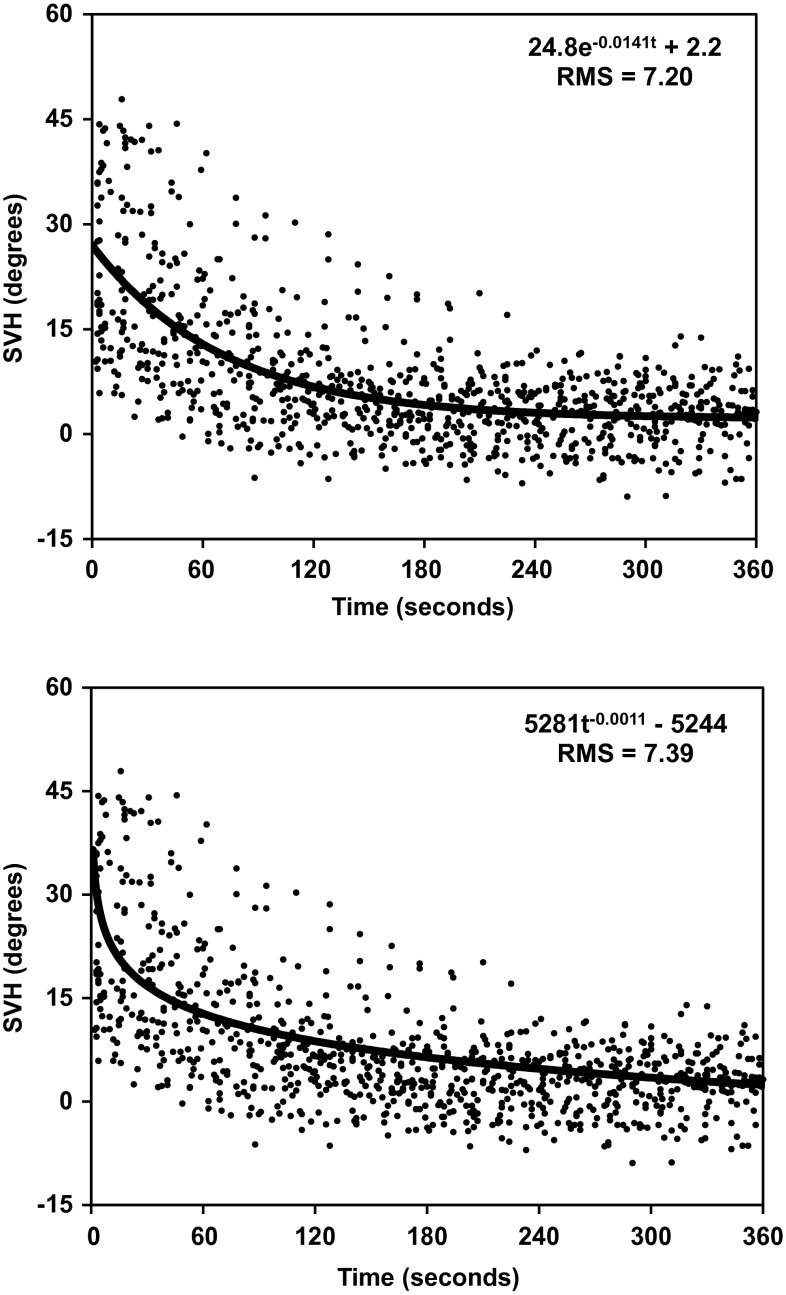
Adaptation of the exponential function (*upper diagram*) and the power function (*lower diagram*) to all settings of the line made by all subjects. Although the two curves clearly have different forms, they are similar in terms of goodness of fit (RMS error)

Curve fitting was also performed on the basis of group means for 15 and 5 time intervals, respectively; in both cases the RMS error was notably greater for the power function than for the exponential function (see Fig. 3). However, when the functions were fit to all 921 data points (Fig. 4), the difference in RMS error between the exponential (RMS = 7.20) and power functions (RMS = 7.39) was negligible.

## Discussion

One reason to mathematically characterize psychological or behavioural memory phenomena is that this might facilitate the identification of underlying neuronal mechanisms; if a particular memory process has a time course similar to that of a biochemical reaction then there is the possibility that the latter is the mechanism behind the former (cf. Tarnow 2008, 2009). Consequently, the following discussion has two major sections. The first relates the present methodology and findings to other studies on the time course for forgetting. The second deals with certain neural mechanisms, which have been extensively studied in rats and monkeys and which could possibly be of significance for the decay of the SVH tilt during centrifugation. In particular, we will focus on the so-called head-direction system and the sense of direction as well as on a few hippocampal processes whose time courses are similar to that found in the present study.

### The form of the forgetting curve

#### On the present findings

The present findings clearly show that for a simple spatial orientation task (adjusting a luminous line in darkness so that it coincides with the perceived horizontal plane) the forgetting of a roll angular displacement is better represented by an exponential function than by a power function; the RMS was smaller for the exponential function in the vast majority of cases.

A simple exponential decay is usually defined by the formula y = Ae^−bt^, where A is the initial value of y (at t = 0); the time constant T is defined as 1/b. As regards the SVH, there is often a small deviation from the true horizontal. In vestibularly healthy subjects, sitting upright in the static 1-g environment, this deviation is rarely greater than 2.5° but it appears to be a comparatively persistent individual characteristic (Tribukait 2006). At an increased G level in a centrifuge this *static* component (i.e. after decay of semicircular canal effects) of the SVH can be greater (Tribukait and Eiken 2006b), possibly because of asymmetries in the graviceptive systems. This must be taken into account when establishing the time course for the decline in the SVH tilt. Thus, when establishing the exponential time course for the decay of the SVH tilt, we use the function SVH = Ae^−bt^ + C, where C represents the asymptote. In several other memory tasks, where an *amount of information* is to be recalled, C < 0 is impossible and C > 0 would imply that a proportion of the initially presented information will remain in memory forever. On the present measurement scale, however, 0° (the horizontal plane of the gondola) does not represent an absolute limit. Rather, the individual asymptote C corresponds to zero in a task where an amount of information is to be recalled. Nevertheless, although the individual asymptote often differed notably from zero, the group mean did not. In addition, in no case did the *initial* value for the exponential function (A + C) exceed the magnitude of the roll tilt of the gondola (66° at 2.5G).

For the sake of comparability with respect to goodness of fit we have used an asymptote, C, also when fitting the power function. This asymptote tended to assume a negative value, often far beyond the physiological range (i.e. it would suggest a sensation of being rotated several revolutions in roll, which is clearly unreasonable in the present experimental situation). Since the power function goes to infinity as t approaches 0 from the right, we have restricted our attention to an interval ranging from 4 s to 360 s (i.e. the interval during which empirical data were obtained). Within this interval the power function gave reasonable values for all individuals. Nevertheless, even if its parameters were not constrained to what might be considered a physiological range, only in a few single cases did the power function provide a better fit (in terms of RMS error) to data than the exponential function.

#### A brief history of the quantitative study of forgetting

In 1682, the physicist and inventor Robert Hooke presented a model of memory that implies a power function for decay (see Hintzman 2003, for an analysis). The systematic experimental study of human forgetting seems to have started 200 years later, namely with the investigations by Ebbinghaus (1885) on his own ability to remember lists of nonsense syllables. He measured the time necessary for re-learning lists at different time intervals—retention intervals—after first having learnt them. Naturally, re-learning a given list required less time than learning it at the first occasion. The time *saved* because of the first learning occasion decreased in a characteristic way with increasing retention intervals (ranging from 21 min to 31 days). Many attempts to mathematically characterize this “savings function” have been made (see Rubin and Wenzel 1996 or White 2001, for references).

Conceivably, the mathematical characteristics of forgetting might differ between memory systems. On the one hand, it seems reasonable that a very simple task, as that employed in the present study, will yield forgetting curves reflecting basic neural mechanisms. On the other hand, the forgetting of complex impressions might be optimized with respect to the odds that a given event will occur again in the natural environment (Anderson and Schooler 1991); an individual’s forgetting of a particular situation would, thus, not be merely a passive process—or a failure phenomenon—but *adapted* to the declining probability of encountering a similar situation again.

A common distinction is that between short-term and long-term memory. In 1973 Wickelgren suggested that this distinction could be made on the basis of the form of the retention function; he had observed that while the exponential function provided a good fit to certain data for retention intervals smaller than 20 s, for intervals ranging between 20 s and 2 years the power function was more adequate. However, in a later publication Wickelgren (1974) refuted the distinction in favour of his “single-trace fragility theory”, stating that the decay of a memory trace is determined by the passage of time as well as by interference according to the function m = λ(1 + βt)^−ψ^e^−πt^. According to this view, the retention curve could be predominated either by the power-function characteristic of time decay or by the exponential-function characteristic of interference, the latter, according to Wickelgren, being the case in many short-term memory tasks.

Whereas the power function has since long possessed a certain status within psychology—in laws regarding the perception of stimulus intensity (Stevens 1970) as well as when it comes to the effects of training (several examples are given by Newell and Rosenbloom 1981)—Wickelgren is often quoted for having introduced it in the study of forgetting. In 1991 Wixted and Ebbesen presented three experiments which differed considerably not only with respect to the time scale but also as regards the nature of the task; in a delayed matching-to-sample task with pigeons the maximum retention interval was 6 s while in a face-recognition test with human test subjects the retention interval ranged between 1 h and 2 weeks. Strikingly, in all cases the power function provided a better fit to data than five alternatives, including the exponential function (see also Wixted and Ebbesen 1997). In the search for regularities and laws Rubin and Wenzel (1996) made adaptations of 105 different 2-parameter functions to 210 sets of forgetting data found in the literature. The power function and the exponential in the square root of time were among the four that could be satisfactorily fit to a wide range of data.

#### The effect of curve fitting to group data

Several authors have pointed out that if there is a substantial inter-individual variability in the time constant for a truly exponential memory decay, then group data may nevertheless be best represented by a power function (Anderson 2001; Anderson and Tweney 1997; Murre and Chessa 2011). This observation has been made also in the study of learning. Heathcote et al. (2000) analyzed 40 data sets representing 475 subjects in 24 skill-acquisition experiments. In more than 80 % of individual cases the exponential function provided a better description of the time course for learning. However, averaging produced a bias in favour of the power function. In the present material, the individual RMS error was on average 31 % greater for the power function than for the exponential function; however, the difference in RMS was negligible if a single curve fit was made to all 921 data points. This confirms that averaging truly exponential forgetting curves may generate a power function.

According to Sikström (1999) forgetting in accordance with the power law would occur also *within* individuals if performance is dependent on multiple memory traces decaying exponentially with different time constants. Thus, on the perceptual or behavioural level exponential forgetting curves would most likely be encountered if the measured quantity corresponds to a basic percept, such as the subjective visual horizontal.

#### Criteria for data sets useful for discriminating between functions

Rubin et al. (1999) have proposed six criteria that would define a data set useful for discriminating between mathematical functions. These are, briefly: (1) there should be nine or more retention intervals. (2) Time values should be precise. (3) The data set should have a large ratio of the most to least amount remembered and a large ratio of the longest to shortest retention intervals. (4) To make the time between presentation and testing unambiguous, each item should be presented only once. (5) The activity that fills the retention intervals should be constant. (6) To prevent generating a group function that is not applicable to individuals, retention functions should be fitted to data of individuals.

Although some of these criteria are perhaps not directly applicable to the stimulus and task of the present study, it might be noted that (1) all individuals made 15 or more settings of the luminous line during the 360-s recording interval, (2) for each setting there is a precise time value, (3) in all cases the ratio between the change in SVH during the recording interval and the RMS was large; the same holds true regarding the ratio between the time values for the last and first settings, (4) the nature of the task used in the present study is such that multiple measurements can be made after one single stimulus, (5) it might be assumed that any mental activity would not considerably influence a basic vestibular percept like the SVH, (6) curve fitting could be performed in every individual case.

Thus, also on the basis of such task-independent criteria it appears that the present methodology, i.e. using a simple visual indicator to quantify the perceptual effects of a vestibular stimulus, could serve as a tool in the study of short-term forgetting. If the decline of the SVH represents a more general mechanism for short-term memory, as could possibly be established by considering inter-individual variability and the degree of correlation across tasks, then the simplicity of the present test paradigm would be useful, for instance, when investigating how pharmacological substances influence short-term memory.

###  Neurophysiological considerations

#### The perceptual representation system

In the present study we have used a psychophysical method to record how a short-term memory for semicircular canal information is reflected in a simple visual percept. It is a point of the method that the SVH is not likely to be influenced by cognitive reasoning; the orientation of a line is among the basic properties of visual stimuli that are analysed in the primary visual cortex, V1 (Hubel and Wiesel 1977, Spillmann 2014). A large number of studies have elucidated the nature of a *non*-*conscious* system devoted to elementary attributes of visual objects or sceneries. This so-called perceptual representation system (Tulving and Schacter 1990) is located early in the cortical processing stream but beyond V1 (see Magnussen and Greenlee 1999, for a review). It is constituted by a number of independent mechanisms, each comprising a separate memory function, which process, in parallel, the different dimensions of visual impressions. A pioneering study on this system was performed by Hegelmaier (1852), who tested his own memory for the length of lines, i.e. a visual attribute, the retention of which is not likely to be influenced by verbal coding strategies. More recent works have investigated the short-term memory for spatial frequency (Magnussen et al. 1990), motion (Magnussen and Greenlee 1992), and—notably—orientation (Magnussen et al. 1998).

The perceived stability of the visual world during changes in roll head position is another phenomenon that seems likely to be attained via such unconscious processing. In humans, ocular counter-torsion—“external compensation” for head tilt—is only vestigial (i.e. it amounts to approximately 10 % of the head-tilt angle); our perception of the visual surroundings as upright also during head tilt with respect to gravity must therefore be dependent on a purely neural mechanism, sometimes denoted “internal compensation” (Bischof 1974; Petrov and Zenkin 1973). As to the localisation in the brain of such vestibular influence on visual perception one possibility is the parietal cortex (Andersen 1997; Bremmer et al. 2002), a region which has important functional connections with the hippocampus (Save et al. 2005).

#### The hippocampus

When considering spatial memory phenomena and the sense of balance a brain region of particular interest is the hippocampal formation. It plays a key role both in spatial orientation (O’Keefe 1990) and in the formation and retrieval of memories, including the linkage of episodes to a spatial context (Burgess et al. 2002; Hayakawa et al. 2015; Kumaran and Maguire 2007; Ventriglia 2008; Wagatsuma and Yamaguchi 2007). The hippocampus receives input from many areas of the cerebral association cortex, e.g. the parietal cortex, whose integrity is significant for several spatial functions (for a review of experimental evidence, see Rolls 1996). The rodent hippocampus is well known for the so-called place cells, a set of neurons, each having its maximum firing rate when the animal is at a certain position in space (O’Keefe 1979). Also in monkeys, single cell recordings have revealed a variety of hippocampal neurons activated in conjunction with spatial stimuli or tasks (for a review, see Rolls 1996). A cell type common in primates is the “view cell”, i.e. pyramidal neurons whose activity is dependent on what part of the surroundings the monkey is looking at irrespectively of its own position (Rolls and O’Mara 1995; Killian et al. 2012; Rolls et al. 1997). It also appears that the firing of view cells is independent of the position of the eye with respect to the head (Georges-Francois et al. 1999). It has been convincingly argued that while the rodent hippocampus contains a map-like representation with neurons representing places where the rat can be, the corresponding system in the primate hippocampus would be devoted to *visual* exploration of the surroundings (Rolls 2006). Nevertheless, a functioning view-cell system must be dependent also on mechanisms for tracking the animal’s position and orientation with respect to the surroundings, i.e. on place cells and head direction cells (see below). Even if these cell types have been studied mainly for the horizontal plane it is tempting to postulate that similar neural mechanisms exist also for the roll (frontal) plane, e.g. when it comes to the ability to perceive the orientation of a line with respect to gravity.

Several lines of research indicate a certain relationship between the hippocampus and the sense of balance. It has even been proposed that the development of the hippocampus during evolution was to a high extent dependent on vestibular information about head movements and about the orientation of the head in the gravitational force field (Smith et al. 2009). More specifically, place cells in the rodent hippocampus can be modulated by vestibular stimulation (Gavrilov et al. 1995; Wiener et al. 1995). In monkeys, there are hippocampal neurons responding to passive whole-body angular displacements even in the absence of visual cues (O’Mara et al. 1994). In humans, stimulation of the semicircular canals by caloric irrigation causes an activation of the hippocampus, as revealed by means of fMRI (Vitte et al. 1996; Suzuki et al. 2001). Bilateral loss of vestibular function disrupts the activity of hippocampal place cells in rodents (Russell et al. 2003, Stackman et al. 2002); in humans it may result in a substantial reduction in the volume of the hippocampus (Brandt et al. 2005). Thus, although neural mechanisms of spatial orientation are difficult to study directly in human subjects, there are indirect evidences as to the significance of the hippocampus for vestibular phenomena also in humans.

#### The head direction system

A navigational system must also be capable of keeping track of the animal’s *direction*. Neurons whose firing rate is a function of the animal’s head direction in the plane of locomotion have been found in several regions of the mammalian brain. These so-called “head direction (HD) cells” are hierarchically organized in a circuit originating in the dorsal tegmental nucleus (Sharp et al. 2001b) and projecting serially via the lateral mammillary nucleus (Blair et al. 1998; Stackman and Taube 1998), anterodorsal thalamus (Taube 1995; Blair and Sharp 1995) and postsubiculum (Ranck 1984; Taube et al. 1990a) to the entorhinal cortex (Sargolini et al. 2006) the latter being a major link between the hippocampus and other regions of the brain (Hayakawa et al. 2015; Zhang et al. 2013). For detailed accounts on the head direction system, the reader should consult reviews by Hartley et al. (2013), Knierim and Hamilton (2011) or Taube (2007).

Head direction cells integrate multimodal information regarding the animal’s movements with respect to the surroundings. The functional integrity of the semicircular canals is critical for the HD system—for the mere generation of a stable HD signal as well as for its continuous updating during head movements. Thus, whereas the properties of HD cells are maintained in darkness, permanent inactivation of the semicircular canals leads to an irreversible disruption of the HD signal (Muir et al. 2004; Stackman and Taube 1997; Stackman et al. 2002). Nevertheless, visual input about external landmarks seems likely to be necessary to avoid the accumulation of errors in the HD system; rotation of a salient visual landmark can induce a corresponding shift in the head direction signal (Taube 1995; Taube et al. 1990b). Thus, the way of functioning of the head direction system reflects an intimate interaction between vision and the sense of balance.

The HD system is believed to constitute a *continuous attractor network* which can perform angular path integration, i.e. integrate the angular-velocity signal from the semicircular canals over time to obtain a measure of angular displacement (Barry and Burgess 2014; Clark and Taube 2012; Sharp et al. 2001a). A representation of angular head velocity is conveyed from the medial vestibular nucleus via the supragenual nucleus (Biazoli et al. 2006) and the nucleus prepositus hypoglossi (Lannou et al. 1984) to the dorsal tegmental nucleus (Brown et al. 2005) (For a review, see Yoder and Taube 2014). In models of this neural integrator, HD cells are interconnected in a circular arrangement, each cell firing maximally for a particular head direction. Neurons with similar preferred head directions have excitatory connections whereas cells which differ greatly in preferred direction inhibit each other. This principle for interconnections gives rise to a self-maintaining activation hill (the attractor state) which corresponds to the HD signal. At a given point in time, the firing pattern can only represent one single head direction, but, in case of a head turn, the system integrates the canal angular-velocity signal over time whereby the activity hill is smoothly moved to a new stable state (Barry and Burgess 2014; Clark and Taube 2012).

The HD system has several characteristics in common with the oculomotor neural integrator (Robinson 1989; see Taube and Bassett 2003, for an analysis). However, in oculomotor integrator neurons, whose firing rate is proportional to the deviation of the eye from its neutral position, there is a tendency to “leak”. In other words, when an animal is gazing in an eccentric direction in darkness, the eyes gradually return to their neutral position, and, in parallel, there is a decreasing firing in oculomotor integrator neurons. Considering the HD system as a ring attractor network, there seems to be no reason to assume that there would be such decay phenomena or any particular neutral direction. Nevertheless, it has been observed that upon removing external visual landmarks or extinguishing the light, the preferred direction of HD cells may drift during several minutes (Goodridge et al. 1998; Taube et al. 1990b). Further, during sensory conflicts, where vision and the sense of balance do not provide unanimous information, the preferred direction of a HD cell was often found to partially shift, its response being a compromise between the information from the two senses (Goodridge et al. 1998; Taube et al. 1990b).

The properties and organization of the HD system have been elucidated mainly via electrophysiological experiments on rats, but HD cells have been identified in several other species, including monkeys (Robertson et al. 1999; Rolls 2006). Studies using fMRI suggest that HD cells exist in the human medial parietal (Baumann and Mattingley 2010) and medial temporal (Vass and Epstein 2013) lobes.

Although the HD system has been investigated almost exclusively for the plane of navigation, it is reasonable to assume that memory processes for the roll (frontal) plane have a related neurophysiologic basis. In both cases it is a matter of semicircular canal information that must be integrated over time to yield an estimate of angular head displacement. Further, the shifts in a HD cell’s preferred direction observed during visuo-vestibular conflicts are reminiscent of the decay in the SVH tilt found during the otolith-semicircular canal conflict in the centrifuge. Finally, the changes in preferred direction of HD cells often occurred during several minutes (Goodridge et al. 1998; Taube et al. 1990b), which is compatible with the slow time course for the SVH observed in the present study.

#### Short-term memory and neuronal dynamics

Short-term memory phenomena are generally believed to depend on neuronal activation, or a change in action potential discharge, persisting after the presentation of a stimulus. Such so-called *persistent neural activation* has been observed in many areas of the cortex as well as in subcortical regions of the brain (see Curtis and Lee 2010 or Major and Tank 2004, for references). The mechanisms by which activity is maintained can, in principle, be intrinsic to individual neurons—as for instance when repeated brief stimuli result in a step-like depolarization via calcium influx at dendrites—as well as related to synaptic plasticity or neural network dynamics (for references, see Curtis and Lee 2010).

The above-mentioned hippocampal mechanisms for spatial orientation, as well as the role of the hippocampus in memory processes, makes it particularly relevant to consider this brain region when discussing the time constant for decay of the SVH tilt during gondola centrifugation. One mechanism that has been much studied in the hippocampus is the temporary increase in synaptic strength denoted short-term potentiation (STP). Anwyl et al. (1988) used in vitro slice preparations for studying STP in area CA1 pyramidal cells of the rodent hippocampus. In certain conditions the time constant for decay of STP was 60–80 s. More recently, it has been reported that interneurons in mice hippocampal slices are capable of performing a slow integration that causes action potential initiation in the distal axon (Sheffield et al. 2011). In these neurons repeated somatic current injections eventually triggered persistent firing, decaying with a time constant of 50–150 s. Thus, these cells were functioning like a leaky integrator. The phenomenon has been observed also in hippocampal slices from rats and it could be elicited with stimulus patterns recorded in vivo in an awake rat, suggesting that it was not an artefact of excessive spiking stimulation (Sheffield et al. 2011). Although these hippocampal decay phenomena have so far not been analyzed with respect to mathematical form (e.g. whether they proceed according to an exponential or a power function), they are both compatible with the time constant found in the present study (T = 85 s).

If the semicircular canal memory phenomenon concerned in the present paper were related to any of these mechanisms, then it would be of a somewhat more general interest regarding human hippocampal functioning, which is difficult to study directly in normal subjects. Possibly, the present test paradigm could be useful in the study of how the hippocampus is influenced by pharmacological substances. In a clinical perspective it should be noted that the roll inclination (swing out) of the gondola constitutes a tangible canal stimulus also for G levels considerably lower than 2.5G, making it feasible to test not only healthy subjects.

## References

[CR1] Andersen RA (1997). Multimodal integration for the representation of space in the posterior parietal cortex. Philos Trans R Soc Lond B Biol Sci.

[CR2] Anderson RB (2001). The power law as an emergent property. Mem Cognit.

[CR3] Anderson JR, Schooler LJ (1991). Reflections of the environment in memory. Psychol Sci.

[CR4] Anderson RB, Tweney ED (1997). Artifactual power curves in forgetting. Mem Cognit.

[CR5] Anwyl R, Lee W-L, Rowan M (1988). The role of calcium in short-term potentiation in the rat hippocampal slice. Brain Res.

[CR6] Aubert H (1861). Eine scheinbare bedeutende Drehung von Objecten bei Neigung des Kopfes nach rechts oder links. Wirchow’s Arch.

[CR7] Baddeley AD (1986). Working memory.

[CR8] Barry C, Burgess N (2014). Neural mechanisms of self-location. Curr Biol.

[CR9] Baumann O, Mattingley JB (2010). Medial parietal cortex encodes perceived heading direction in humans. J Neurosci.

[CR10] Biazoli CE, Goto M, Campos AM, Canteras NS (2006). The supragenual nucleus: a putative relay station for ascending vestibular signs to head direction cells. Brain Res.

[CR11] Bischof N, Kornhuber HH (1974). Optic-vestibular orientation to the vertical. Handbook of sensory physiology, vol VI/2.

[CR12] Blair HT, Sharp PE (1995). Anticipatory firing of anterior thalamic head direction cells: evidence for a thalamocortical circuit that computes head direction in the rat. J Neurosci.

[CR13] Blair HT, Cho J, Sharp PE (1998). Role of the lateral mammillary nucleus in the rat head direction circuit: a combined single unit recording and lesion study. Neuron.

[CR14] Brandt T, Dieterich M (1999). The vestibular cortex. Its locations, functions, and disorders. Ann N Y Acad Sci.

[CR15] Brandt T, Schautzer F, Hamilton DA, Brüning R, Markowitsch HJ, Kalla R, Darlington C, Smith P, Strupp M (2005). Vestibular loss causes hippocampal atrophy and impaired spatial memory in humans. Brain.

[CR16] Bremmer F, Klam F, Duhamel J-R, Hamed SB, Graf W (2002). Visual-vestibular interactive responses in the macaque ventral intraparietal area (VIP). Eur J Neurosci.

[CR17] Brown JE, Card JP, Yates BJ (2005). Polysynaptic pathways from the vestibular nuclei to the lateral mammillary nucleus of the rat: substrates for vestibular input to head direction cells. Exp Brain Res.

[CR18] Burgess N, Maguire EA, O’Keefe J (2002). The human hippocampus and spatial and episodic memory. Neuron.

[CR19] Cassel JC, Cassel D, Manning L (2012). From Augustine of Hippo’s memory systems to our modern taxonomy in cognitive psychology and neuroscience of memory: a 16-century nap of intuition before light of evidence. Behav Sci (Basel).

[CR20] Clark BJ, Taube JS (2012). Vestibular and attractor network basis of the head direction cell signal in subcortical circuits. Front Neural Circuits.

[CR21] Curtis EC, Lee D (2010). Beyond working memory: the role of persistent activity in decision making. Trends Cogn Sci.

[CR22] Dai MJ, Curthoys IS, Halmagyi GM (1989). Linear acceleration perception in the roll plane before and after unilateral vestibular neurectomy. Exp Brain Res.

[CR23] Danziger K (2008). Marking the mind: a history of memory.

[CR24] Ebbinghaus H (1885). Über das Gedächtnis. Untersuchungen zur experimentellen Psychologie.

[CR25] Fabbro F, Aglioti SM, Bergamasco M, Clarici A, Panksepp J (2015). Evolutionary aspects of self- and world consciousness in vertebrates. Front Hum Neurosci.

[CR26] Gavrilov VV, Wiener SI, Berthoz A (1995). Enhanced hippocampal theta EEG during whole body rotations in awake restrained rats. Neurosci Lett.

[CR27] Georges-Francois P, Rolls ET, Robertson RG (1999). Spatial view cells in the primate hippocampus: allocentric view not head direction or eye position or place. Cereb Cortex.

[CR28] Glasauer S (1993) Human spatial orientation during centrifuge experiments: Nonlinear interaction of semicircular canals and otoliths. In: Krejcova H, Jerabek J (eds) Proceedings of the XVIIth Barany society meeting, Prague 1992, pp 48–52

[CR29] Goodridge JP, Dudchenko PA, Worboys KA, Golob EJ, Taube JS (1998). Cue control and head direction cells. Behav Neurosci.

[CR30] Guedry FE, Rupert AH, McGrath BJ, Oman CM (1992). The dynamics of spatial orientation during complex and changing linear and angular acceleration. J Vest Res.

[CR31] Harris DC (1998). Nonlinear least-squares curve fitting with Microsoft Excel Solver. J Chem Ed.

[CR32] Hartley T, Lever C, Burgess N, O’Keefe J (2013). Space in the brain: how the hippocampal formation supports spatial cognition. Philos Trans R Soc Lond B Biol Sci.

[CR33] Hayakawa H, Samura T, Kamijo TC, Sakai Y, Aihara T (2015). Spatial information enhanced by non-spatial information in hippocampal granule cells. Cogn Neurodyn.

[CR34] Heathcote A, Brown S, Mewhort DJK (2000). The power law repealed: the case for an exponential law of practice. Psychonom Bull Rev.

[CR35] Hegelmaier F (1852). Über das Gedächtnis für Linearanschauungen. Archiv für Physiologisch Heilkunde.

[CR36] Hintzman DL (2003). Robert Hooke’s model of memory. Psychonom Bull Rev.

[CR37] Hubel DH, Wiesel TN (1977). The Ferrier lecture: functional architecture of macaque monkey visual cortex. Proc R Soc Lond B.

[CR38] Israël I, Bronstein AM, Kanayama R, Faldon M, Gresty MA (1996). Visual and vestibular factors influencing vestibular “navigation”. Exp Brain Res.

[CR39] Katz PS (2007). Evolution and development of neural circuits in invertebrates. Curr Opin Neurobiol.

[CR40] Killian NJ, Jutras MJ, Buffalo EA (2012). A map of visual space in the primate entorhinal cortex. Nature.

[CR41] Knierim JJ, Hamilton DA (2011). Framing spatial cognition: neural representations of proximal and distal frames of reference and their roles in navigation. Physiol Rev.

[CR42] Kumaran D, Maguire EA (2007). Match mismatch processes underlie human hippocampal responses to associative novelty. J Neurosci.

[CR43] Lannou J, Cazin L, Precht W, Le Taillanter M (1984). Responses of prepositus hypoglossi neurons to optikinetic and vestibular stimulations in the rat. Brain Res.

[CR44] Logie RH, Zucco GM, Baddeley AD (1990). Interference with visual short-term memory. Acta Psychol.

[CR45] Lopez C, Blanke O, Mast FW (2012). The human vestibular cortex revealed by coordinate-based activation likelihood estimation meta-analysis. Neuroscience.

[CR46] Magnussen S, Greenlee MW (1992). Retention and disruption of motion information in visual short-term memory. J Exp Psychol Learn Mem Cogn.

[CR47] Magnussen S, Greenlee MW (1999). The psychophysics of perceptual memory. Psychol Res.

[CR48] Magnussen S, Greenlee MW, Asplund R, Dyrnes S (1990). Perfect short-term memory for periodic patterns. Eur J Cogn Psychol.

[CR49] Magnussen S, Idås E, Holst-Myhre S (1998). Representation of orientation and spatial frequency in perception and memory: a choice reaction-time analysis. J Exp Psychol Hum Percept Perform.

[CR50] Major G, Tank D (2004). Persistent neural activity: prevalence and mechanisms. Curr Opin Neurobiol.

[CR51] Makovski T, Jiang YV (2008). Proactive interference from items previously stored in visual working memory. Mem Cognit.

[CR52] Mergner T, Rumberger A, Becker W (1996). Is perceived angular displacement the time integral of perceived angular velocity?. Brain Res Bull.

[CR53] Mittelstaedt H (1991). The role of the otoliths in the perception of the orientation of self and world to the vertical. Zool Jb Physiol.

[CR54] Mizraji E, Pomi A, Valle-Lisboa JC (2009). Dynamic searching in the brain. Cogn Neurodyn.

[CR55] Muir GM, Carey JP, Hirvonen TP, Minor LB, Taube JS (2004). Head direction cell activity is unstable following plugging of the semicircular canals in the freely-moving chinchilla. Soc Neurosci Abstr.

[CR56] Müller GE (1916). Über das Aubertsche Phänomen. Z Sinnesphysiol.

[CR57] Murdock BB, Cook CD (1960). On fitting the exponential. Psychol Rep.

[CR58] Murre JMJ, Chessa AG (2011). Power laws from individual differences in learning and forgetting: mathematical analyses. Psychon Bull Rev.

[CR59] Nairne JS (2002). Remembering over the short-term: the case against the standard model. Annu Rev Psychol.

[CR60] Newell A, Rosenbloom PS, Anderson JR (1981). Mechanisms of skill acquisition and the law of practice. Cognitive skills and their acquisition.

[CR61] O’Keefe J (1979). A review of the hippocampal place cells. Prog Neurobiol.

[CR62] O’Keefe J (1990). A computational theory of the hippocampal cognitive map. Prog Brain Res.

[CR63] O’Mara SM, Rolls ET, Berthoz A, Kesner RP (1994). Neurons responding to whole-body motion in the primate hippocampus. J Neurosci.

[CR64] Pellegrino JW, Siegel AW, Dhawan M (1976). Short-term retention of pictures and words as a function of type of distraction and length of delay interval. Mem Cognit.

[CR65] Petrov AP, Zenkin GM (1973). Torsional eye movements and constancy of the visual field. Vision Res.

[CR66] Ranck JB (1984). Head-direction cells in the deep cell layers of the dorsal presubiculum in freely-moving rats. Soc Neurosci Abstr.

[CR67] Ricker TJ, Vergauwe E, Cowan N (2014) Decay theory of immediate memory: from Brown (1958) to today (2014). Q J Exp Psychol [Epub ahead of print]. 10.1080/17470218.2014.914546 10.1080/17470218.2014.914546 PMC4241183 24853316

[CR68] Robertson RG, Rolls ET, Georges-Francois P, Panzeri S (1999). Head direction cells in the primate pre-subiculum. Hippocampus.

[CR69] Robinson DA (1989). Integrating with neurons. Annu Rev Neurosci.

[CR70] Rolls ET, Ishikawa K, McGaugh JL, Sakata H (1996). The representation of space in the primate hippocampus, and its role in memory. Brain processes and memory.

[CR71] Rolls ET (2006). Neurophysiological and computational analyses of the primate presubiculum, subiculum and related areas. Behav Brain Res.

[CR72] Rolls ET, O’Mara SM (1995). View-responsive neurons in the primate hippocampal complex. Hippocampus.

[CR73] Rolls ET, Robertson RG, Georges-Francois P (1997). Spatial view cells in the primate hippocampus. Eur J Neurosci.

[CR74] Rubin DC, Wenzel AE (1996). One hundred years of forgetting: a quantitative description of retention. Psychol Rev.

[CR75] Rubin DC, Hinton S, Wenzel A (1999). The precise time course of retention. J Exp Psychol.

[CR76] Russell NA, Horii A, Smith PF, Darlington CL, Bilkey DK (2003). Long-term effects of permanent vestibular lesions on hippocampal spatial firing. J Neurosci.

[CR77] Sargolini F, Fyhn M, Hafting T, McNaughton BL, Witter MP, Moser MB, Moser EI (2006). Conjunctive representation of position, direction, and velocity in entorhinal cortex. Science.

[CR78] Sarnat HB, Netsky MG (2002). When does a ganglion become a brain? Evolutionary origin of the central nervous system. Semin Pediatr Neurol.

[CR79] Save E, Paz-Villagran V, Alexinsky T, Poucet B (2005). Functional interaction between the associative parietal cortex and hippocampal place cell firing in the rat. Eur J Neurosci.

[CR80] Sharp PE, Blair HT, Cho J (2001). The anatomical and computational basis of the rat head-direction cell signal. Trends Neurosci.

[CR81] Sharp PE, Tinkelman A, Cho J (2001). Angular velocity and head direction signals recorded from the dorsal tegmental nucleus of Gudden in the rat: implications for path integration in the Head Direction cell circuit. Behav Neurosci.

[CR82] Sheffield MEJ, Best TK, Mensh BD, Kath WL, Spruston N (2011). Slow integration leads to persistent action potential firing in distal axons of coupled interneurons. Nat Neurosci.

[CR83] Sikström S (1999) Power function forgetting curves as an emergent property of biologically plausible neural networks model. Int J Psychol. Special Issue: Short-term/working memory 34 (5–6):460–464

[CR84] Silverberg N, Buchanan L (2005). Verbal mediation and memory for novel figural designs: a dual interference study. Brain Cogn.

[CR85] Smith PF, Brandt T, Strupp M, Darlington CL, Zheng Y (2009). Balance before reason in rats and humans. Ann N Y Acad Sci.

[CR86] Spillmann L (2014). Receptive fields of visual neurons: the early years. Perception.

[CR87] Squire LR (2004). Memory systems of the brain: a brief history and current perspective. Neurobiol Learn Mem.

[CR88] Stackman RW, Taube JS (1997). Firing properties of head direction cells in rat anterior thalamic neurons: dependence upon vestibular input. J Neurosci.

[CR89] Stackman RW, Taube JS (1998). Firing properties of rat lateral mammillary single units: head direction, head pitch, and angular head velocity. J Neurosci.

[CR90] Stackman RW, Clark AS, Taube JS (2002). Hippocampal spatial representations require vestibular input. Hippocampus.

[CR91] Stevens SS (1970). Neural events and the psychophysical law. Science.

[CR92] Stockwell CW, Guedry FE (1970). The effect of semicircular canal stimulation during tilting on the subsequent perception of the visual vertical. Acta Otolaryngol.

[CR93] Suzuki M, Kitano H, Ito R, Kitanishi T, Yazawa Y, Ogawa T, Shiino A, Kitajima K (2001). Cortical and sub-cortical vestibular response to caloric stimulation detected by functional magnetic resonance imaging. Brain Res Cogn Brain Res.

[CR94] Swanson LW (2000). What is the brain?. Trends Neurosci.

[CR95] Tarnow E (2008). Response probability and response time: a straight line, the Tagging/Retagging interpretation of short-term memory, an operational definition of meaningfulness and short term memory time decay and search time. Cogn Neurodyn.

[CR96] Tarnow E (2009). Short term memory may be the depletion of the readily releasable pool of presynaptic neurotransmitter vesicles of a metastable long term memory trace pattern. Cogn Neurodyn.

[CR97] Taube JS (1995). Head direction cells recorded in the anterior thalamic nuclei of freely moving rats. J Neurosci.

[CR98] Taube JS (2007). The head direction signal: origins and sensory-motor integration. Annu Rev Neurosci.

[CR99] Taube JS, Bassett JP (2003). Persistent neural activity in head direction cells. Cereb Cortex.

[CR100] Taube JS, Muller RU, Ranck JB (1990). Head-direction cells recorded from the postsubiculum in freely moving rats. I. Description and quantitative analysis. J Neurosci.

[CR101] Taube JS, Muller RU, Ranck JB (1990). Head-direction cells recorded from the postsubiculum in freely moving rats. II. Effects of environmental manipulations. J Neurosci.

[CR102] Taylor JG (2007). On the neurodynamics of the creation of consciousness. Cogn Neurodyn.

[CR103] Tribukait A (2006). Subjective visual horizontal in the upright posture and asymmetry in roll-tilt perception: independent measures of vestibular function. J Vest Res.

[CR104] Tribukait A, Eiken O (2006). Changes in the perceived head transversal plane and the subjective visual horizontal induced by Coriolis stimulation during gondola centrifugation. J Vest Res.

[CR105] Tribukait A, Eiken O (2006). Roll-tilt perception during gondola centrifugation: influence of steady-state acceleration (G) level. Aviat Space Env Med.

[CR106] Tulving E, Schacter DL (1990). Priming and human memory systems. Science.

[CR107] Udo de Haes HA, Schöne H (1970). Interaction between statolith organs and semicircular canals on apparent vertical and nystagmus. Acta Otolaryngol.

[CR108] Van Beuzekom AD, Medendorp WP, Van Gisbergen JAM (2001). The subjective vertical and the sense of self orientation during active body tilt. Vision Res.

[CR109] Vass LK, Epstein RA (2013). Abstract representations of location and facing direction in the human brain. J Neurosci.

[CR110] Ventriglia F (2008). The engram formation and the global oscillations of CA3. Cogn Neurodyn.

[CR111] Vitte E, Derosier C, Caritu Y, Berthoz A, Hasboun D, Soulié D (1996). Activation of the hippocampal formation by vestibular stimulation: a functional magnetic resonance imaging study. Exp Brain Res.

[CR112] Von Holst E, Grisebach E (1951). Einfluss des Bogengangssystems auf die “subjective Lotrechte” beim Menschen. Naturwissenschaften.

[CR113] Wagatsuma H, Yamaguchi Y (2007). Neural dynamics of the cognitive map in the hippocampus. Cogn Neurodyn.

[CR114] Werner G (2009). Consciousness related neural events viewed as brain state space transitions. Cogn Neurodyn.

[CR115] White KG (2001). Forgetting functions. Anim Learn Behav.

[CR116] Wickelgren WA (1973). The long and the short of memory. Psychol Bull.

[CR117] Wickelgren WA (1974). Single-trace fragility theory of memory dynamics. Mem Cognit.

[CR118] Wiener SI, Korshunov VA, Garcia R, Berthoz A (1995). Inertial, substratal and landmark cue control of hippocampal CA1 place cell activity. Eur J Neurosci.

[CR119] Wixted JT, Ebbesen EB (1991). On the form of forgetting. Psychol Sci.

[CR120] Wixted JT, Ebbesen EB (1997). Genuine power curves in forgetting: a quantitative analysis of individual subject forgetting functions. Mem Cognit.

[CR121] Yoder RM, Taube JS (2014). The vestibular contribution to the head direction signal and navigation. Front Integr Neurosci.

[CR122] Young LR, Geiger SR (1984). Perception of the body in space: mechanisms. Handbook of physiology III/1.

[CR123] Zhang SJ, Ye J, Couey JJ, Witter M, Moser EI, Moser MB (2013). Functional connectivity of the entorhinal–hippocampal space circuit. Philos Trans R Soc Lond B Biol Sci.

